# 7*H*-1-Benzofuro[2,3-*b*]carbazole

**DOI:** 10.1107/S1600536811039018

**Published:** 2011-09-30

**Authors:** R. Panchatcharam, V. Dhayalan, A. K. Mohanakrishnan, G. Chakkaravarthi, V. Manivannan

**Affiliations:** a Centre for Research and Development, PRIST University, Vallam, Thanjavur 613 403, Tamil Nadu, India; b Department of Organic Chemistry, University of Madras, Guindy Campus, Chennai 600 025, India; c Department of Physics, CPCL Polytechnic College, Chennai 600 068, India

## Abstract

In the title compound, C_18_H_11_NO, the carbazole and benzofuran rings are almost co-planar, making a dihedral angle of 3.31 (3)°. The crystal structure is stabilized by weak C—H⋯π inter­actions.

## Related literature

For the biological activity of carbazole derivatives, see: Ramsewak *et al.* (1999 ▶); Diaz *et al.* (2002 ▶); Zhang *et al.* (2004 ▶). For the structures of closely related compounds, see: Chakkaravarthi *et al.* (2008*a* ▶,*b* ▶).
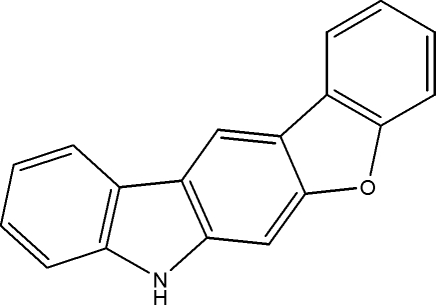


## Experimental

### 

#### Crystal data

C_18_H_11_NO
*M* *_r_* = 257.28
Orthorhombic, 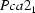

*a* = 26.087 (3) Å
*b* = 5.9585 (8) Å
*c* = 7.8146 (10) Å
*V* = 1214.7 (3) Å^3^
*Z* = 4
Mo *K*α radiation
μ = 0.09 mm^−1^
*T* = 295 K
0.24 × 0.22 × 0.20 mm

#### Data collection

Bruker Kappa APEXII diffractometer
Absorption correction: multi-scan (*SADABS*; Sheldrick, 1996 ▶) *T*_min_ = 0.979, *T*_max_ = 0.983
6591 measured reflections
2700 independent reflections
2331 reflections with *I* > 2σ(*I*)
*R*_int_ = 0.022

#### Refinement

*R*[*F* ^2^ > 2σ(*F* ^2^)] = 0.033
*wR*(*F* ^2^) = 0.084
*S* = 1.05
2700 reflections
182 parameters
H-atom parameters constrained
Δρ_max_ = 0.13 e Å^−3^
Δρ_min_ = −0.13 e Å^−3^
Absolute structure: Flack (1983 ▶), 1086 Friedel pairs
Flack parameter: 0.2 (15)

### 

Data collection: *APEX2* (Bruker, 2004 ▶); cell refinement: *SAINT* (Bruker, 2004 ▶); data reduction: *SAINT*; program(s) used to solve structure: *SHELXS97* (Sheldrick, 2008 ▶); program(s) used to refine structure: *SHELXL97* (Sheldrick, 2008 ▶); molecular graphics: *PLATON* (Spek, 2009 ▶); software used to prepare material for publication: *SHELXL97*.

## Supplementary Material

Crystal structure: contains datablock(s) global, I. DOI: 10.1107/S1600536811039018/vm2122sup1.cif

Structure factors: contains datablock(s) I. DOI: 10.1107/S1600536811039018/vm2122Isup2.hkl

Supplementary material file. DOI: 10.1107/S1600536811039018/vm2122Isup3.cml

Additional supplementary materials:  crystallographic information; 3D view; checkCIF report

## Figures and Tables

**Table 1:** Hydrogen-bond geometry (Å, °) *Cg*2, *Cg*3 and *Cg*4 are the centroids of the N1/C1/C6/C7/C12, C1–C6 and C7–C12 rings, respectively.

*D*—H⋯*A*	*D*—H	H⋯*A*	*D*⋯*A*	*D*—H⋯*A*
N1—H1⋯*Cg*4^i^	0.86	2.91	3.530 (2)	130
C5—H5⋯*Cg*2^ii^	0.93	2.70	3.449 (2)	138
C11—H11⋯*Cg*3^i^	0.93	2.79	3.526 (2)	137

Symmetry codes: (i) 
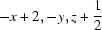
; (ii) 
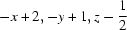
.

**Fig. 1.**
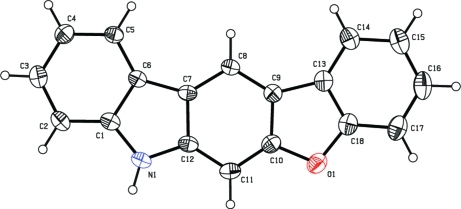
The molecular structure of the title compound with atom labels and 30% probability displacement ellipsoids for non-H atoms.

## supplementary crystallographic information

### Comment

Carbazole derivatives exhibit anti-inflammatory and antimutagenic activities (Ramsewak *et al.*, 1999). A carbazole ring is easily functionalized and covalently linked to other molecules (Diaz *et al.*, 2002). This enables its use as a convenient building block for the design and synthesis of molecular glasses, which are widely studied as components of electroactive and photoactive materials (Zhang *et al.*, 2004).

We herewith report the crystal structure of the title compound (Fig. 1). The geometric parameters agree well with those of similar reported structures (Chakkaravarthi *et al.*, 2008*a*, 2008*b*). The benzofuran moiety (C9/C13-C18/O1/C10) is almost co-planar [dihedral angle 3.31 (3)°] with the carbazole ring (C1-C12/N1). The crystal structure is stabilized by weak intermolecular C-H···π interactions (Table 1).

### Experimental

To a solution of tert-butyl-3-(2,2-di(ethoxycarbonyl)vinyl)-2-(bromomethyl) -1H-indole-1-carboxylate (0.5 g, 1.04 mmol) in dry 1,2-dichloroethane (15 mL), anhydrous ZnBr_2_ (0.05 g, 0.22 mmol) and benzo[b]furan (0.13 mL, 1.27 mmol) were added. It was then stirred at room temperature for 2 h under N_2_ atmosphere. The solvent was removed and the residue was quenched with ice-water (50 mL) containing 1 mL of conc. HCl, extracted with chloroform (2 x 10 mL) and dried (Na_2_SO_4_). Removal of solvent followed by flash column chromatography purification (n-hexane/ethyl acetate 80:20) led to the isolation of title compound as a colourless crystal suitable for X-Ray diffraction quality.

### Refinement

All H atoms were positioned geometrically, with C—H = 0.93Å and N—H = 0.86Å and allowed to ride on their parent atoms, with U_iso_(H) = 1.2 U_eq_(C,N).

### Crystal data

**Table:**

C_18_H_11_NO	*F*(000) = 536
*M**_r_* = 257.28	*D*_x_ = 1.407 Mg m^−^^3^
Orthorhombic, *P**c**a*2_1_	Mo *K*α radiation, λ = 0.71073 Å
Hall symbol: P 2c -2ac	Cell parameters from 2246 reflections
*a* = 26.087 (3) Å	θ = 1.6–28.3°
*b* = 5.9585 (8) Å	µ = 0.09 mm^−^^1^
*c* = 7.8146 (10) Å	*T* = 295 K
*V* = 1214.7 (3) Å^3^	Block, colourless
*Z* = 4	0.24 × 0.22 × 0.20 mm

### Data collection

**Table:**

Bruker Kappa APEXII diffractometer	2700 independent reflections
Radiation source: fine-focus sealed tube	2331 reflections with *I* > 2σ(*I*)
graphite	*R*_int_ = 0.022
ω and φ scans	θ_max_ = 28.3°, θ_min_ = 1.6°
Absorption correction: multi-scan (*SADABS*; Sheldrick, 1996)	*h* = −33→34
*T*_min_ = 0.979, *T*_max_ = 0.983	*k* = −7→7
6591 measured reflections	*l* = −7→10

### Refinement

**Table:**

Refinement on *F*^2^	Hydrogen site location: inferred from neighbouring sites
Least-squares matrix: full	H-atom parameters constrained
*R*[*F*^2^ > 2σ(*F*^2^)] = 0.033	*w* = 1/[σ^2^(*F*_o_^2^) + (0.0437*P*)^2^ + 0.0541*P*] where *P* = (*F*_o_^2^ + 2*F*_c_^2^)/3
*wR*(*F*^2^) = 0.084	(Δ/σ)_max_ < 0.001
*S* = 1.05	Δρ_max_ = 0.13 e Å^−^^3^
2700 reflections	Δρ_min_ = −0.13 e Å^−^^3^
182 parameters	Extinction correction: *SHELXL*, Fc^*^=kFc[1+0.001xFc^2^λ^3^/sin(2θ)]^-1/4^
0 restraints	Extinction coefficient: 0.0083 (13)
Primary atom site location: structure-invariant direct methods	Absolute structure: Flack (1983), 1086 Friedel pairs
Secondary atom site location: difference Fourier map	Flack parameter: 0.2 (15)

### Fractional atomic coordinates and isotropic or equivalent isotropic displacement parameters (Å^2^)

**Table:**

	*x*	*y*	*z*	*U*_iso_*/*U*_eq_	
C1	0.95422 (6)	0.1951 (2)	0.5133 (2)	0.0412 (3)	
C2	0.90187 (6)	0.2333 (3)	0.5252 (2)	0.0481 (4)	
H2	0.8802	0.1313	0.5790	0.058*	
C3	0.88330 (6)	0.4286 (3)	0.4541 (2)	0.0526 (4)	
H3	0.8484	0.4587	0.4602	0.063*	
C4	0.91550 (6)	0.5822 (3)	0.3733 (2)	0.0491 (4)	
H4	0.9017	0.7122	0.3262	0.059*	
C5	0.96747 (6)	0.5437 (2)	0.3624 (2)	0.0414 (3)	
H5	0.9889	0.6474	0.3093	0.050*	
C6	0.98745 (5)	0.3475 (2)	0.43203 (19)	0.0370 (3)	
C7	1.03818 (5)	0.2503 (2)	0.43841 (19)	0.0366 (3)	
C8	1.08582 (5)	0.3196 (2)	0.37727 (18)	0.0386 (3)	
H8	1.0899	0.4576	0.3233	0.046*	
C9	1.12697 (6)	0.1751 (2)	0.4000 (2)	0.0412 (3)	
C10	1.11930 (6)	−0.0328 (2)	0.4815 (2)	0.0439 (4)	
C11	1.07375 (6)	−0.1066 (3)	0.5477 (2)	0.0464 (4)	
H11	1.0703	−0.2431	0.6042	0.056*	
C12	1.03323 (6)	0.0405 (2)	0.5230 (2)	0.0403 (3)	
C13	1.18108 (6)	0.1825 (3)	0.3568 (2)	0.0458 (4)	
C14	1.21349 (6)	0.3394 (3)	0.2832 (2)	0.0525 (4)	
H14	1.2009	0.4763	0.2444	0.063*	
C15	1.26512 (6)	0.2873 (4)	0.2688 (3)	0.0617 (5)	
H15	1.2874	0.3903	0.2192	0.074*	
C16	1.28401 (7)	0.0836 (4)	0.3275 (3)	0.0666 (6)	
H16	1.3188	0.0533	0.3169	0.080*	
C17	1.25258 (7)	−0.0744 (3)	0.4009 (2)	0.0624 (5)	
H17	1.2652	−0.2110	0.4403	0.075*	
C18	1.20141 (6)	−0.0194 (3)	0.4131 (2)	0.0508 (4)	
N1	0.98259 (5)	0.0150 (2)	0.57102 (18)	0.0443 (3)	
H1	0.9706	−0.0965	0.6283	0.053*	
O1	1.16449 (4)	−0.15463 (18)	0.48849 (17)	0.0557 (3)	

### Atomic displacement parameters (Å^2^)

**Table:**

	*U*^11^	*U*^22^	*U*^33^	*U*^12^	*U*^13^	*U*^23^
C1	0.0493 (8)	0.0409 (7)	0.0336 (8)	−0.0090 (6)	−0.0002 (7)	−0.0032 (6)
C2	0.0450 (8)	0.0557 (9)	0.0434 (9)	−0.0134 (7)	0.0036 (7)	−0.0009 (8)
C3	0.0419 (8)	0.0659 (11)	0.0501 (10)	−0.0015 (7)	0.0016 (7)	−0.0052 (8)
C4	0.0511 (8)	0.0487 (8)	0.0474 (10)	0.0039 (7)	−0.0041 (8)	−0.0009 (8)
C5	0.0470 (7)	0.0383 (7)	0.0389 (8)	−0.0052 (6)	−0.0007 (7)	−0.0001 (6)
C6	0.0421 (7)	0.0381 (7)	0.0309 (7)	−0.0070 (5)	−0.0014 (6)	−0.0026 (6)
C7	0.0472 (7)	0.0313 (6)	0.0312 (7)	−0.0050 (6)	−0.0010 (6)	−0.0018 (6)
C8	0.0453 (7)	0.0350 (7)	0.0354 (8)	−0.0054 (6)	0.0002 (6)	0.0013 (6)
C9	0.0446 (7)	0.0402 (7)	0.0387 (9)	−0.0026 (6)	−0.0001 (6)	−0.0059 (6)
C10	0.0506 (8)	0.0386 (7)	0.0425 (9)	0.0033 (6)	−0.0040 (7)	−0.0048 (6)
C11	0.0596 (9)	0.0343 (7)	0.0452 (9)	−0.0023 (6)	−0.0016 (7)	0.0021 (7)
C12	0.0513 (8)	0.0347 (7)	0.0349 (8)	−0.0080 (6)	−0.0018 (6)	−0.0020 (6)
C13	0.0454 (8)	0.0519 (9)	0.0402 (9)	−0.0008 (7)	−0.0024 (7)	−0.0100 (7)
C14	0.0483 (9)	0.0633 (10)	0.0459 (10)	−0.0072 (7)	−0.0015 (8)	−0.0075 (8)
C15	0.0482 (9)	0.0839 (13)	0.0529 (12)	−0.0106 (9)	0.0043 (9)	−0.0151 (10)
C16	0.0451 (9)	0.0932 (15)	0.0616 (13)	0.0072 (10)	0.0007 (8)	−0.0240 (11)
C17	0.0550 (9)	0.0677 (10)	0.0645 (14)	0.0147 (9)	−0.0031 (9)	−0.0143 (10)
C18	0.0500 (8)	0.0540 (9)	0.0485 (11)	0.0034 (7)	−0.0025 (7)	−0.0109 (8)
N1	0.0505 (7)	0.0391 (6)	0.0432 (8)	−0.0106 (5)	0.0034 (6)	0.0053 (6)
O1	0.0558 (6)	0.0471 (6)	0.0643 (9)	0.0090 (5)	−0.0031 (6)	−0.0019 (5)

### Geometric parameters (Å, °)

**Table:**

C1—N1	1.380 (2)	C10—C11	1.369 (2)
C1—C2	1.388 (2)	C10—O1	1.3856 (18)
C1—C6	1.407 (2)	C11—C12	1.387 (2)
C2—C3	1.377 (2)	C11—H11	0.9300
C2—H2	0.9300	C12—N1	1.3817 (19)
C3—C4	1.394 (2)	C13—C14	1.385 (2)
C3—H3	0.9300	C13—C18	1.386 (2)
C4—C5	1.378 (2)	C14—C15	1.387 (2)
C4—H4	0.9300	C14—H14	0.9300
C5—C6	1.391 (2)	C15—C16	1.388 (3)
C5—H5	0.9300	C15—H15	0.9300
C6—C7	1.445 (2)	C16—C17	1.374 (3)
C7—C8	1.3940 (19)	C16—H16	0.9300
C7—C12	1.4202 (19)	C17—C18	1.378 (2)
C8—C9	1.388 (2)	C17—H17	0.9300
C8—H8	0.9300	C18—O1	1.387 (2)
C9—C10	1.407 (2)	N1—H1	0.8600
C9—C13	1.452 (2)		
			
N1—C1—C2	129.30 (14)	O1—C10—C9	110.99 (13)
N1—C1—C6	108.61 (13)	C10—C11—C12	113.97 (14)
C2—C1—C6	122.09 (14)	C10—C11—H11	123.0
C3—C2—C1	117.21 (15)	C12—C11—H11	123.0
C3—C2—H2	121.4	N1—C12—C11	128.43 (13)
C1—C2—H2	121.4	N1—C12—C7	108.07 (13)
C2—C3—C4	121.72 (15)	C11—C12—C7	123.50 (14)
C2—C3—H3	119.1	C14—C13—C18	118.94 (15)
C4—C3—H3	119.1	C14—C13—C9	135.24 (15)
C5—C4—C3	120.78 (15)	C18—C13—C9	105.78 (14)
C5—C4—H4	119.6	C13—C14—C15	118.36 (17)
C3—C4—H4	119.6	C13—C14—H14	120.8
C4—C5—C6	118.98 (14)	C15—C14—H14	120.8
C4—C5—H5	120.5	C14—C15—C16	120.92 (18)
C6—C5—H5	120.5	C14—C15—H15	119.5
C5—C6—C1	119.21 (13)	C16—C15—H15	119.5
C5—C6—C7	133.90 (13)	C17—C16—C15	121.69 (16)
C1—C6—C7	106.87 (13)	C17—C16—H16	119.2
C8—C7—C12	120.09 (13)	C15—C16—H16	119.2
C8—C7—C6	133.30 (13)	C16—C17—C18	116.36 (18)
C12—C7—C6	106.59 (12)	C16—C17—H17	121.8
C9—C8—C7	117.52 (12)	C18—C17—H17	121.8
C9—C8—H8	121.2	C17—C18—C13	123.72 (17)
C7—C8—H8	121.2	C17—C18—O1	124.33 (16)
C8—C9—C10	119.61 (13)	C13—C18—O1	111.91 (14)
C8—C9—C13	134.72 (14)	C1—N1—C12	109.77 (12)
C10—C9—C13	105.67 (13)	C1—N1—H1	125.1
C11—C10—O1	123.74 (14)	C12—N1—H1	125.1
C11—C10—C9	125.27 (14)	C10—O1—C18	105.64 (12)
			
N1—C1—C2—C3	−179.31 (16)	C8—C7—C12—N1	179.51 (13)
C6—C1—C2—C3	−0.1 (2)	C6—C7—C12—N1	−2.04 (16)
C1—C2—C3—C4	0.0 (3)	C8—C7—C12—C11	−1.0 (2)
C2—C3—C4—C5	−0.3 (3)	C6—C7—C12—C11	177.49 (15)
C3—C4—C5—C6	0.6 (2)	C8—C9—C13—C14	3.5 (3)
C4—C5—C6—C1	−0.7 (2)	C10—C9—C13—C14	−176.81 (17)
C4—C5—C6—C7	177.28 (15)	C8—C9—C13—C18	−179.06 (17)
N1—C1—C6—C5	179.83 (13)	C10—C9—C13—C18	0.64 (18)
C2—C1—C6—C5	0.5 (2)	C18—C13—C14—C15	0.1 (2)
N1—C1—C6—C7	1.33 (16)	C9—C13—C14—C15	177.25 (18)
C2—C1—C6—C7	−178.01 (15)	C13—C14—C15—C16	−0.4 (3)
C5—C6—C7—C8	0.4 (3)	C14—C15—C16—C17	0.4 (3)
C1—C6—C7—C8	178.59 (16)	C15—C16—C17—C18	0.0 (3)
C5—C6—C7—C12	−177.74 (17)	C16—C17—C18—C13	−0.3 (3)
C1—C6—C7—C12	0.44 (16)	C16—C17—C18—O1	−177.71 (17)
C12—C7—C8—C9	1.1 (2)	C14—C13—C18—C17	0.3 (3)
C6—C7—C8—C9	−176.87 (15)	C9—C13—C18—C17	−177.67 (16)
C7—C8—C9—C10	0.3 (2)	C14—C13—C18—O1	177.98 (13)
C7—C8—C9—C13	179.98 (16)	C9—C13—C18—O1	0.04 (19)
C8—C9—C10—C11	−2.1 (2)	C2—C1—N1—C12	176.60 (17)
C13—C9—C10—C11	178.14 (16)	C6—C1—N1—C12	−2.68 (17)
C8—C9—C10—O1	178.64 (14)	C11—C12—N1—C1	−176.56 (16)
C13—C9—C10—O1	−1.11 (17)	C7—C12—N1—C1	2.94 (17)
O1—C10—C11—C12	−178.66 (15)	C11—C10—O1—C18	−178.14 (15)
C9—C10—C11—C12	2.2 (2)	C9—C10—O1—C18	1.13 (17)
C10—C11—C12—N1	178.80 (15)	C17—C18—O1—C10	176.98 (17)
C10—C11—C12—C7	−0.6 (2)	C13—C18—O1—C10	−0.71 (18)

### Hydrogen-bond geometry (Å, °)

**Table:**

Cg2, Cg3 and Cg4 are the centroids of the N1/C1/C6/C7/C12, C1–C6 and C7–C12 rings, respectively.

**Table:**

*D*—H···*A*	*D*—H	H···*A*	*D*···*A*	*D*—H···*A*
N1—H1···Cg4^i^	0.86	2.91	3.530 (2)	130
C5—H5···Cg2^ii^	0.93	2.70	3.449 (2)	138
C11—H11···Cg3^i^	0.93	2.79	3.526 (2)	137

Symmetry codes: (i) −*x*+2, −*y*, *z*+1/2; (ii) −*x*+2, −*y*+1, *z*−1/2.
